# Abdominal Inflammatory Myofibroblastic Tumor: A Rare Case

**DOI:** 10.5005/jp-journals-10018-1196

**Published:** 2016-12-01

**Authors:** Fatih Tastekin, Mustafa Ersoy, Tuncer Temel, Safak Meric Ozgenel, Funda Canaz, Aysegül Özakyol

**Affiliations:** 1 Department of Internal Medicine, Eskisehir Osmangazi University, Eskisehir, Turkey; 2 Department of Gastroenterology, Eskisehir Osmangazi University Eskisehir, Turkey; 3 Department of Pathology, Eskisehir Osmangazi University, Eskisehir, Turkey

**Keywords:** Abdominal mass, Inflammatory myofibroblastic tumor, Inflammatory pseudotumor.

## Abstract

An inflammatory myofibroblastic tumor, also known as inflammatory pseudotumor, is a rare neoplasm characterized by myofibroblastic spindle and inflammatory cells that cause masses in many sites of body. It is often benign, but in some cases neoplastic transformation has been reported as a result of aggressive growing. In our case, an inflammatory myofibroblastic tumor was reported by biopsy of a 25 × 15 cm abdominal mass.

**How to cite this article:**

Tastekin F, Ersoy M, Temel T, Ozgenel SM, Canaz F, Özakyol A. Abdominal Inflammatory Myofibroblastic Tumor: A Rare Case. Euroasian J Hepato-Gastroenterol 2016;6(2):183-185.

## INTRODUCTION

Inflammatory myofibroblastic tumor is a disorder that cause masses in many sites of body and that is not still to be proven whether it is an inflammatory course or a true neoplasm.^1^ It is often benign, but in some cases neoplastic transformation has been reported as a result of aggressive growing. Other names of this entity in the literature are inflammatory pseudotumor, pseudosarcomatous myofibroblastic proliferation, pseudosarcomatous myofibroblastic tumor, pseudosarcomatous reactive response, atypical myofibroblastic tumor, atypical fibromixoid tumor, plasma cell granuloma, and bladder nodular fasciitis.

## CASE REPORT

**Fig. 1:**
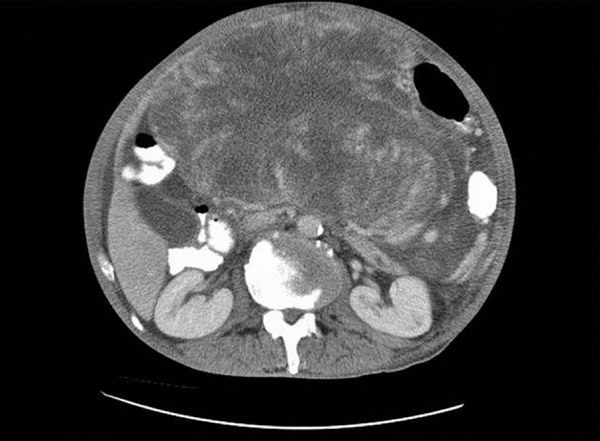
Inflammatory mass by MRI

**Fig. 2:**
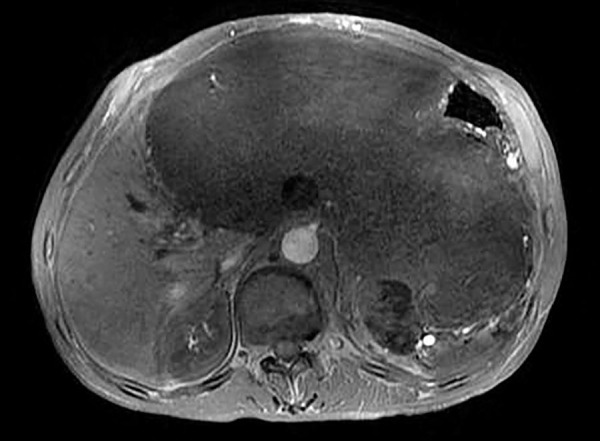
Inflammatory mass by MRI

**Fig. 3:**
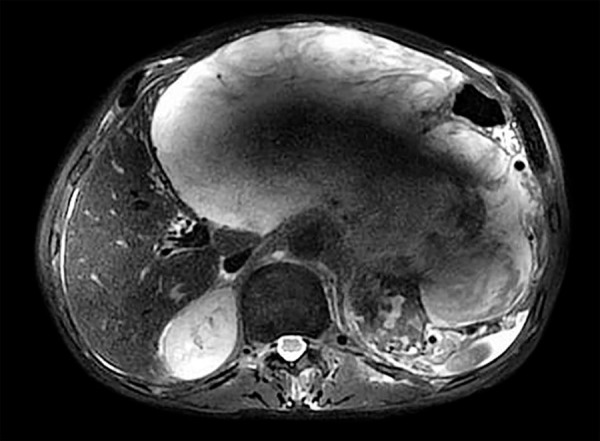
Inflammatory mass by MRI

**Fig. 4:**
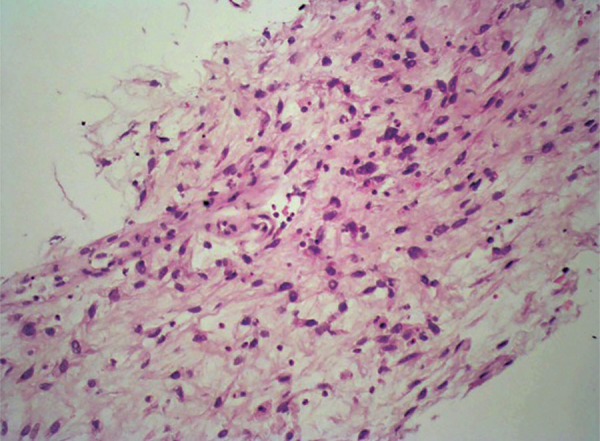
Histopathological assessment of inflammatory mass

A 60-year-old man has been examined due to complaints like oral intake disorder and weight loss, and an abdominal mass was detected. He was referred to our department (Eskişehir Osmangazi University, Medical Faculty Hospital, Gastroenterology Clinic) by the suspicion of cancer derived liver. A 25 × 15 × 24 cm mass, which has an undistinguishable tissue origin, was observed on dynamic magnetic resonance imaging (MRI) (Figs 1 to 3). The mass caused compression on stomach and the other tissues within the abdomen. A peripheral parenteral nutrition was started because the stomach collapsed. We performed an ultrasound-guided biopsy and determined a mesenchymal spindle cell tumor (Fig. 4). So, a curative or a palliative surgery was decided by consultation of medical oncology and general surgery department. The mass could not be excised due to invasion during the operation. A jejunostomy has been performed for enteral nutrition, and the patient was referred to medical oncology for further therapy.

## DISCUSSION

An inflammatory myofibroblastic tumor is often referred as an inflammatory pseudotumor. It is a rare neoplasm characterized by myofibroblastic spindle and inflammatory cells and is particularly located in soft tissues. It is often benign, but in some cases, neoplastic transformation has been reported as a result of aggressive growing.^2^ Infection, trauma, irradiation, and autoimmune ground are considered in disorder’s etiology, but etiology is still not clear.^3^ Symptoms are varied based on involved body part. However, abdominal discomfort, dyspepsia, oral intake disorder, weight loss, and mass with palpation may be seen in cases with abdominal involvement, such as the case in our patient. When we examine patterns macroscopically, they are benign, restricted, and not encapsulated; also nodular and diffuse growing patterns are observed. If we investigate it microscopically, spindle cell proliferation and mixed types of inflammatory cells are there in collagenated loose stroma. Immunohistochemical investigation contributes to confirmation of diagnosis. Vimentin, Smooth Muscle Actin (SMA), and Cluster of differentiation 68 (CD68) are positive. In addition, anaplastic lymphoma kinase (ALK) reveals cytoplasmic staining in myofibroblastic cells.^4^ Ultrasonography, especially computed tomography and MRI, is necessary for diagnosis. Most common involved body part is lung, but involvement of larynx, spleen, breast, bladder, central nervous system, pancreas, orbita, mesentery, prostate, salivary glands, testes, and skin have been reported.^5,6^ First choice of treatment, if available, is complete resection in most patients; if not, radiotherapy and steroid use is recommended. The other treatment choices are immunomodulation (cyclosporine A), chemotherapy (methotrexate, azatiopurine, chlorambucil, cyclophosphamide, iphosphamide, vincristin, dactinomycin), and antimetabolite regimen.^2^ Early diagnosis and total excision are significant in good prognosis. Although pathogenic organisms are reported in most inflammatory myofibroblastic tumor cases, resection should be always considered due to presence of malignant transformation in some cases.

## CONCLUSION

We evaluated a rare inflammatory myofibroblastic tumor in our case. The tumor was large and had an interesting pathogenesis. Therefore, we decided to report it.
